# High prevalence of HIV and non‐communicable disease (NCD) risk factors in rural KwaZulu‐Natal, South Africa

**DOI:** 10.1002/jia2.25012

**Published:** 2017-10-24

**Authors:** Alastair van Heerden, Ruanne V. Barnabas, Shane A. Norris, Lisa K. Micklesfield, Heidi van Rooyen, Connie Celum

**Affiliations:** ^1^ Human and Social Development (HSD) Human Sciences Research Council Pietermaritzburg South Africa; ^2^ MRC/Wits Developmental Pathways for Health Research Unit Faculty of Health Sciences University of the Witwatersrand Johannesburg South Africa; ^3^ Departments of Global Health and Medicine University of Washington Seattle WA USA

**Keywords:** NCDs, community screening, HIV, multiple comorbidities

## Abstract

**Introduction:**

South Africa faces epidemics of HIV and non‐communicable diseases (NCDs). The aim of this study was to characterize the prevalence of non‐communicable disease risk factors and depression, stratified by HIV status, in a community with a high burden of HIV.

**Methods:**

We conducted a home‐based HIV counselling and testing study in KwaZulu‐Natal, South Africa between November 2011 and June 2012. Contiguous households were approached and all adults ≥18 years old were offered an HIV test. During follow‐up visits in January 2015, screening for HIV, depression, obesity, blood glucose, cholesterol and blood pressure were conducted using point‐of‐care tests.

**Results:**

Of the 570 participants located and screened; 69% were female and 33% were HIV‐positive. NCD risk factor prevalence was high in this sample; 71% were overweight (body mass index (BMI) 25 to 29.9 kg/m^2^) or obese (BMI≥30 kg/m^2^), 4% had hyperglycaemia (plasma glucose >11.0 mmol/l/200 mg/dl), 33% had hypertension (HTN, >140/90 mmHg), 20% had hyperlipidaemia (low density cholesterol >5.2 mmol/l/193.6 mg/dl) and 12% had major depressive symptoms (nine item Patient Health Questionnaire ≥10). Of the 570 participants, 87% had one or more of HIV, hyperglycaemia, HTN, hyperlipidaemia and/or depression. Over half (56%) had two or more. Older age and female gender were significantly associated with the prevalence of both HIV infection and NCD risk factors. Around 80% of both HIV‐positive and negative persons had one of the measured risk factors (i.e. obesity, hyperglycaemia, hyperlipidaemia, HTN), or depression.

**Conclusions:**

In a community‐based sample of adults in KwaZulu‐Natal, South Africa, the prevalence of both HIV infection and NCD risk factors were high. This study is among the first to quantify the substantial burden of NCD risk factors and depression in this non‐clinic based population.

## 1 Introduction

The incidence of non‐communicable diseases (NCDs) continues to rise globally 1, 2, 3, 4, 5. Over 85% of NCD‐associated deaths occurring in low‐ or middle‐income countries (LMIC) 6. Cardiometabolic and pulmonary disease, cancer, and mental health disorders are among the most prevalent NCDs in LMICs 7. The estimated hypertension (HTN) prevalence in South Africa is 31.8% 8. At a national level, 7.7% of the individuals sampled were found to be pre‐diabetic and 7.9% diabetic based on Haemoglobin A1c (HbA1c) levels (cut‐off ≥6.5%). Elevated LDL cholesterol levels were found in 21.4% of individuals in the same sample (cut‐off ≥5.2 mmol/l/193.6 mg/dl). Body mass index (BMI) was significantly different by sex with a mean BMI of 23.2 kg/m^2^ for males and 28.9 kg/m^2^ for females, with 30.8% of males and 64.1% of females in the overweight or obese range. In the general populations NCDs accounted for almost 30% of deaths in Africa in 2012 9.

Infectious diseases are already major health threats in sub‐Saharan Africa 10. Eastern and Southern Africa are home to 19 of the 36.7 million (52%) people living with HIV 11. HIV increases the risk of cardiovascular disease (CVD), in part through proinflammatory‐mediated mechanisms. HIV and NCDs are syndemic and associated with an Combination HIV prevention, prevention of mother to child transmission and population level testing and treatment programmes are effective strategies for reducing HIV‐ and AIDS‐related incidence and mortality, whereas at the same time increasing life expectancy 12. Approximately 18 million people are on chronic anti‐retroviral therapy (ART) for HIV 11, which allows HIV‐positive persons to live to healthy middle‐age with normal life‐expectancy. HIV and NCDs are syndemic and associated with an increase in NCDs among HIV‐positive persons; including CVD, HTN, diabetes (DM), hyperlipidaemia, overweight/obesity and cervical cancer 9, 13, 14. Furthermore, these syndemics may emerge also due to socio‐environmental factors that impact biology to increase the prevalence of co‐morbidities.

The co‐occurrence of HIV and NCDs presents many challenges. Comorbidity increases the complexity of patient management, often leading to poorer health outcomes and increased health care costs 15, 16. Comorbidity also poses a huge financial and health burden on households, especially in sub‐Saharan Africa where poverty levels are high with rising cost for healthcare utilization 1, 5, 10. These trends present a growing concern in LMICs as the pattern of NCD comorbidity may be different to that which has been described in high‐income countries, due in part to the younger age distribution of persons living with HIV 5.

The objective of this study was to characterize the prevalence of NCD risk factors and depression, stratified by HIV status, in a high HIV prevalence setting 17, 18. These data can inform integrated community‐based health screening programmes.

## 2 Methods

### 2.1 Study design and participants

This cross‐sectional, single site study was conducted in rural and peri‐urban South Africa following a prospective, multi‐country, cohort study of a package of HIV testing, referral to care and follow‐up visits to increase engagement in HIV care 19. The selected communities are characterized by high unemployment, low income and an HIV prevalence of 28% 20. Geographic Information System (GIS) mapping was used to enumerate all households in the community and participants recruited through home visits. All households from the original study were revisited. Participants were consented and offered the standard home‐based counselling and testing service (HBCT). They were also informed about the additional HBCT‐NCD data collection activity aimed at providing a more general picture of their health and wellbeing, and invited to participate. If a participant was unavailable at the time of the first visit, the household was visited a maximum of two more times. The study was approved by both Human Sciences Research Council Research Ethics Committee (REC: 1/26/05/11) and the University of Washington Institutional Review Board (48733).

### 2.2 Procedures

Lay counsellors were trained by an enrolled nurse in HIV testing, anthropometric measurement, and point of care screening for NCD risk. Once enrolled by the lay counsellors, participants completed a comprehensive interviewer‐led health questionnaire including information on demographics, mental health, working memory, NCD risk factors, chronic conditions and health‐care utilization. Among the socio‐demographic variables were items on sex, date of birth, educational attainment, employment status and household assets. Both continuous and categorical age variables were derived from date of birth. Employment status was a binary variable with all those answering that they were employed then providing their occupation. Socio‐economic status tertiles were derived using a household asset‐based score 21. Following the survey, height and weight were collected from each participant. Height was measured without shoes to the nearest 0.1 cm (0.04 inches) using a Seca Stadiometer (Seca, Birmingham, UK). Weight was measured on an electronic scale to the nearest 0.1 kg (0.22 pounds). Blood pressure and pulse were measured in accordance with the American Heart Association recommendations using Omron HBP‐1300‐E devices (Omron Global, Tokyo, Japan). Participants were seated and three consecutive readings were averaged. A push button lancet safety needle was used to draw blood from the finger for simultaneous HIV testing using the Determine HIV one to two rapid test (Alere Medical Co Ltd., Waltham, MA, USA) and random plasma glucose (RPG) and low‐density lipoprotein (LDL) using the point‐of‐careET‐202 Easy Touch (GC) device and test strips. Results along with appropriate interpretation and counselling messages were delivered to participants and recorded. Repeat HIV testing was offered to all participants who tested negative at the first HBCT visit and who reported their current HIV status as negative. On accepting the offer of HIV test, participants were tested, counseled and referred to care in alignment with the South African National HIV Counselling and Testing Policy Guidelines (May 2015). Participants who tested HIV‐positive during the primary study visit in 2015 were offer a repeat test. Those who accepted were retested and those who did not had their status recorded as HIV positive. All survey and measurement data were captured using the Mobenzi Researcher mobile Android application (Mobenzi Researcher, Durban, South Africa).

### 2.3 Outcomes

The primary outcomes included prevalence of HIV, obesity, HTN, hyperglycaemia, hyperlipidaemia and depression. Cut‐offs for each were based on accepted international norms. A BMI of 25 to 29.9 kg/m^2^ was classified as overweight, whereas a BMI of 30 to 34.9 kg/m^2^ was classified as obese Class I and BMI of ≥35 kg/m^2^ as obese Class II 22. Non‐fasting glucose concentrations of greater than 11.0 mmol/l (200 mg/dl) 23 and LDL levels of greater than 5.2 mmol/l (193.6 mg/dl) were considered abnormal. HTN Stage 1 (Systolic 140–159 mmHg or Diastolic 90–99 mmHg) and Stage 2 (Systolic ≥160 mmHg or Diastolic ≥100 mmHg) were defined based on classifications from the Joint National Committee on Prevention, Detection, Evaluation, and Treatment of High Blood Pressure 24. A short form Patient Health Questionnaire (PHQ‐9) score of ten or more was used as the standard cut‐off indicating major depressive symptoms 25. The PHQ‐9 has been previously validated for use among all ages of the South African population 26, 27, 28.

### 2.4 Statistical analysis

Data were first assessed to characterize prevalence distributions, stratified by HIV status, using descriptive statistics. Next, multiple unadjusted bivariate logistic regression models were fit to the data to examine each disease risk factor and sex, age, employment, education level, HIV and depression. Adjusted multivariate odds ratios were then calculated for each disease risk factor controlling for these demographic variables. We chose to include these covariates as they were known a priori to be associated with NCD risk. All statistical tests were two‐sided and were considered statistically significant at α=0.05. Data were analyzed using SPSS version 22. Finally, to determine the comorbidity of HIV and NCD risk factors, we computed simple co‐occurrence frequencies of HIV and one, two or three additional NCD risk factors all disaggregated by age and sex.

## 3 Results

### 3.1 Sample Characterization

A total of 591 participants were located and screened for enrolment in the study. Of these participants, 570 (96.4%) enrolled into the study. Fifteen people refused participation with the primary reason given being that they did not have time to participate and six were not eligible. Two‐thirds were female and 33.3% of participants in this study tested HIV positive. Overall 71.2% of participants were overweight or obese, 3.9% had elevated RPG, 33.3% presented with HTN, 20.0% had elevated LDL levels and 11.9% screened positive for depression (Table 1). Five percent of the sample reported having suicidal thoughts several times in the past two weeks. When asked if they had ever been diagnosed as diabetic, 41 (7.2%) of the participants agreed with 35 (85.4%) currently on medication. A quarter of the sample report having been diagnosed with HTN and 80% were currently taking treatment for their HTN.

**Table 1:** Unadjusted frequency of disease risk in sample (N=570)

	Missing (n)	Above risk threshold (n)	Percent above risk threshold (%)
Overweight or obese	11	398	71%
Overweight	11	131	23%
Obese class I	11	130	23%
Obese class II	11	137	25%
Random plasma glucose (RPG)	4	22	4%
Hypertension stage 1 or 2	0	190	33%
Hypertension stage 1	0	125	22%
Hypertension stage 2	0	65	11%
LDL	0	113	20%
Depression	0	68	12%

A BMI of 25 to 29.9 kg/m^2^ was classified as overweight, whereas a BMI of 30 to 34.9 kg/m^2^ was classified as obese Class I and BMI of ≥35 kg/m^2^ as obese Class II. Depression was assesses with a PHQ‐9 score ≥10. RPG >11.0 mmol/l (200 mg/dl). LDL >5.2 mmol/l (193.6 mg/dl). Hypertension Stage 1 (Systolic 140–159 mmHg or Diastolic 90–99 mmHg) and Stage 2 (Systolic ≥160 mmHg or Diastolic ≥100 mmHg). BMI, body mass index

Sex, age and educational attainment differed between people living with HIV and those who were not, whereas socio‐economic‐status and employment were similar between the two groups (Table 2). Females were more likely to be HIV positive, and overweight or obese with the mean BMI of males being 25.1 kg/m^2^ and for females 33.0 kg/m^2^ (t(557)=11.96, *p* < 0.01). Among all participants, mental health was found to decline with age as shown by increasing proportion of participants with PHQ≥10 in the older age groups (Table 3).

**Table 2:** Frequency of disease risk and demographic profile by HIV status

	HIV‐negative persons n (%)	HIV‐positive persons n (%)	All persons
Total	381 (100)	189 (100)	570 (100)
Overweight or obese			
Underweight	9 (2)	8 (4)	17 (3)
Healthy	108 (28)	36 (19)	144 (25)
Overweight	82 (22)	49 (27)	131 (23)
Obese class I and II	175 (46)	92 (50)	267 (47)
Missing	7 (2)	4 (2)	11 (2)
Hypertension**			
Healthy	108 (28)	70 (37)	178 (31)
Prehypertension	129 (34)	73 (39)	202 (35)
Hypertension: stage 1	94 (25)	31 (16)	125 (22)
Hypertension: stage 2	50 (13)	15 (8)	65 (11)
Hyperglycaemia**			
Glucose ≤11.0 mmol/l (200 mg/dl)	362 (95)	185 (98)	547 (96)
Glucose >11.0 mmol/l (200 mg/dl)	19 (5)	4 (2)	23 (4)
Hyperlipidaemia			
LDL ≤5.2 mmol/l (193.6 mg/dl)	305 (80)	152 (80)	457 (80)
LDL >5.2 mmol/l (193.6 mg/dl)	76 (20)	37 (20)	113 (20)
Depression**			
PHQ‐9 <10	327 (86)	175 (93)	502 (88)
PHQ‐9 *≥*10	54 (14)	14 (7)	68 (12)
Sex**			
Male	142 (37)	36 (19)	178 (31)
Female	239 (63)	153 (81)	392 (69)
Age**			
18 to 25	80 (21)	19 (10)	99 (17)
26 to 35	78 (21)	64 (34)	142 (25)
36 to 45	43 (11)	62 (34)	105 (18)
46 to 65	102 (27)	40 (21)	142 (25)
66+	78 (20)	4 (2)	82 (14)
Employed			
No	293 (77)	144 (76)	437 (77)
Yes	88 (23)	45 (24)	133 (23)
Socio‐economic status			
Tertile one	114 (30)	52 (27)	166 (29)
Tertile two	150 (39)	85 (45)	235 (41)
Tertile three	117 (31)	52 (28)	269 (47)
Education**			
No primary and completed primary	111 (29)	37 (20)	148 (26)
Completed secondary	105 (28)	79 (42)	184 (32)
Completed high school or more	133 (35)	67 (35)	200 (35)
Missing	32 (8)	6 (3)	38 (7)

Chi‐square test of significance.

**p < 0.01.

**Table 3:** Bivariate and multivariate logistic regressions of disease risk and demographics characteristics

	HIV+		Random plasma glucose		Hypertension		LDL		BMI (≥25 kg/m^2^)		Depression (PHQ ≥10)	
	Unadjusted OR (95% CI)	Adjusted OR (95% CI)	Unadjusted (95% CI)	Adjusted (95% CI)	Unadjusted (95% CI)	Adjusted (95% CI)	Unadjusted (95% CI)	Adjusted (95% CI)	Unadjusted (95% CI)	Adjusted (95% CI)	Unadjusted (95% CI)	Adjusted (95% CI)
Sex												
Male (ref)												
Female	2.5 (1.7 to 3.8)	2.7 (1.7 to 4.3)	3.0 (0.9 to 10.3)	2.2 (0.6 to 8.2)	1.4 (1.0 to 2.1)	1.3 (0.9 to 2.0)	1.2 (0.9 to 1.8)	1.1 (0.8 to 1.7)	6.6 (4.4 to 9.8)	6.1 (4.0 to 9.4)	1.1 (0.6 to 1.9)	1.0 (0.5 to 1.8)
Age												
18 to 25 (ref)												
26 to 35	3.9 (2.1 to 7.2)	3.7 (1.9 to 7.0)	0.7 (0.0 to 11.0)	0.6 (0.0 to 9.8)	1.4 (0.7 to 2.8)	1.2 (0.6 to 2.4)	1.0 (0.6 to 1.7)	1.0 (0.6 to 1.8)	2.3 (1.3 to 3.9)	1.8 (1.0 to 3.3)	1.1 (0.3 to 4.9)	1.0 (0.2 to 4.3)
36 to 45	7.0 (3.7 to 13.4)	6.8 (3.4 to 13.6)	1.8 (0.2 to 20.5)	1.8 (0.2 to 20.5)	2.3 (1.1 to 4.7)	2.1 (1.0 to 4.4)	1.7 (1.0 to 3.0)	1.7 (0.9 to 3.0)	2.8 (1.6 to 5.1)	2.4 (1.2 to 4.7)	2.2 (0.6 to 8.7)	1.3 (0.3 to 5.7)
46 to 65	1.9 (1.0 to 3.5)	1.6 (0.8 to 3.4)	8.0 (1.0 to 63.3)	9.4 (1.0 to 86.6)	6.1 (3.2 to 11.7)	6.0 (2.9 to 12.6)	2.5 (1.4 to 4.2)	2.2 (1.2 to 4.1)	3.9 (2.2 to 6.8)	4.0 (1.9 to 8.2)	5.7 (1.7 to 19.8)	2.1 (0.6 to 8.1)
66+	0.2 (0.1 to 0.8)	0.2 (0.0 to 0.6)	8.9 (1.1 to 73.7)	7.0 (0.6 to 76.2)	8.0 (4.0 to 16.3)	9.9 (4.3 to 22.9)	2.3 (1.3 to 4.3)	2.5 (1.2 to 5.0)	4.1 (2.1 to 8.1)	3.5 (1.5 to 8.2)	19.0 (5.6 to 65.4)	4.6 (1.2 to 17.8)
Employed												
No (ref)												
Yes	1.0 (0.7 to 1.6)	0.7 (0.4 to 1.1)	0.7 (0.2 to 2.2)	1.1 (0.4 to 3.7)	1.3 (0.9 to 1.9)	2.0 (1.3 to 3.2)	0.9 (0.6 to 1.3)	1.0 (0.7 to 1.6)	0.9 (0.6 to 1.4)	1.3 (0.8 to 2.1)	0.3 (0.2 to 0.8)	0.6 (0.3 to 1.5)
Education												
Completed primary or less (ref)												
Completed secondary	2.3 (1.4 to 3.6)	1.6 (0.9 to 2.8)	0.8 (0.3 to 2.2)	1.3 (0.4 to 4.2)	0.7 (0.4 to 1.1)	1.2 (0.7 to 2.0)	0.8 (0.5 to 1.2)	1.0 (0.6 to 1.6	0.7 (0.5 to 1.2)	1.0 (0.6 to 1.9)	0.2 (0.1 to 0.4)	0.4 (0.2 to 0.7)
Completed high school or more	1.5 (0.9 to 2.4)	0.9 (0.5 to 1.6)	0.4 (0.1 to 1.5)	1.7 (0.4 to 7.2)	0.5 (0.3 to 0.7)	1.3 (0.7 to 2.4)	6.2 (0.4 to 1.0)	1.0 (0.6 to 1.8)	0.7 (0.4 to 1.1)	1.2 (0.7 to 2.4)	0.0 (0.0 to 0.1)	0.1 (0.0 to 0.1)
R^2^		0.3		0.1		0.2		0.1		0.2		0.3

BMI, body mass index.

**Figure 1:**
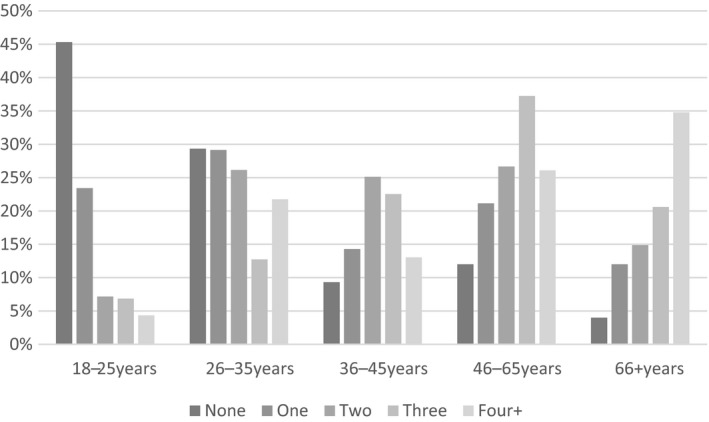
Percentage of sample, stratified by age, with none, one, or more of HIV, hyperlipidaemia, hypertension, hyperglycaemia and depression.

Figure 1 presents an overview of the number of participants with none, one or more of HIV, hyperlipidaemia, HTN, hyperglycaemia and depression. Thirteen percent of the sample was found to have none of the above with 45% of 18‐ to 24‐year olds falling in this category and 4% of participants 66 and older.

### 3.2 Characteristics and NCD risk of HIV‐positive persons

Significantly more female than males tested positive for HIV. HIV was most prevalent in the 26‐ to 45‐year age range, whereas other NCD risk factors typically increased with age. People living with HIV were found to have rates of HTN, hyperglycaemia and hyperlipidaemia similar or somewhat lower than people living without HIV. Without controlling for age and sex, overweight and obesity were more prevalent among people living with HIV.

### 3.3 Characteristics and NCD risk of HIV‐negative persons

**Figure 2:**
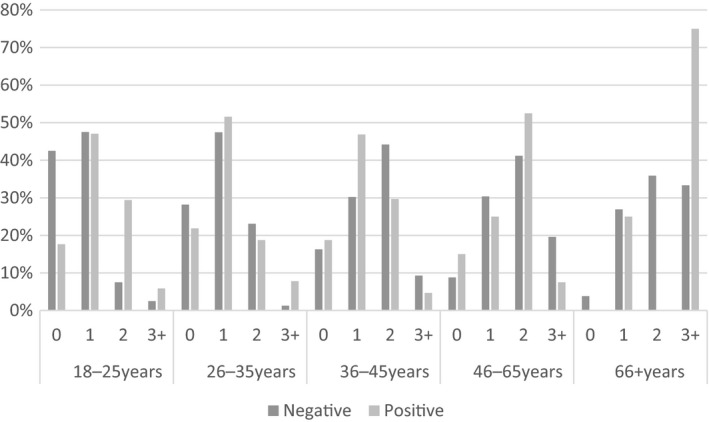
Percentage of people, stratified by age and HIV status, with none, one, or more of hyperlipidaemia, hypertension, hyperglycaemia and depression.

HIV‐negative persons were more likely to be male and older, with 20% being in the 66 years and over age category. While more HIV‐negative persons were of a healthy weight than people living with HIV, two thirds were still overweight or obese. HTN occurred frequently in the HIV‐negative sample and depression was more common. Hyperlipidaemia was equally common among people with and without HIV. The co‐occurrence of multiple NCD risk factors was similar among people living with HIV and HIV‐negative persons (Figure 2).

### 3.4 Adjusted models of disease risk and demographic characterization

Table 3 presents both bivariate unadjusted and multivariate adjusted logistic models for each disease and risk factor after adjusting for age, sex, education and employment. Adjusting for these covariates, females were more than twice as likely to test HIV positive compared to men; however, this sex difference was not significant for any of the cardiometabolic risk factors or depression. Higher risk of HTN and elevated LDL levels was observed in older participants as compared to the reference age group of 18 to 25 years. Depression was found to be twice as likely in the 46‐ to 65‐year age group (OR 1.96; CI 1.01 to 3.82) and three times as likely in the 66 year and older group (OR 2.95; CI 1.36 to 6.41) when compared with 18‐ to 25‐year olds. The only outcome with a discernible association with employment was elevated blood pressure, with those who were employed being twice as likely to be hypertensive compared to those not working (OR 2.03; CI 1.27 to 3.24). Educational attainment was only found to be significantly associated with depression scores. In both the unadjusted and adjusted models, education was protective and associated with significantly lower odds of depression.

## 4 Discussion

The increasing contribution of NCDs to global disease burden was the driver behind the World Health Organization (WHO) 25 × 25 target which aims to reduce global mortality attributable to NCDs by 25% by 2025 29, 30. While the WHO estimates that 42% of the South African population is hypertensive, 32% have elevated levels of LDL and 11% with elevated blood glucose levels 27, these data found the prevalence of HTN to be 33.3%, LDL 19.8% and elevated blood glucose 3.9%. While study limitations restrict direct comparison to national datasets, obesity, HTN and elevated LDL levels appear to present a significant disease burden in this community. These high rates are particularly concerning in this community as over a third are HIV‐positive persons and many already are on chronic long‐term antiretroviral treatment. The presence of these additional cardiometabolic and mental health risk factors are likely to complicate patient management as this population ages, particularly if left unmanaged.

HIV has traditionally been associated with underweight. While the stigma persists, these data found no association between underweight status and HIV status, but rather that many of sample who were HIV positive were overweight or obese. This observation supports the need for widespread education for maintaining a healthy weight, regardless of HIV status 31.

Adjusted regression models also showed that depression is highest among the elderly. These data suggest that older individuals are at significant risk of poor mental health, overweight and obesity, HTN and elevated LDL. Similar findings of elevated NCD risk factors among the elderly have been reported previously in the literature 32, 33. One explanation for why little attention has been paid to HIV and NCDs among older adults is that the initial HIV response focused on mothers and their children. Older adults were not seen as an urgent priority as they were assumed to be at lower risk of HIV acquisition. With the effectiveness of modern ARTs meaning life expectancy of persons with HIV now mirrors that of the general population the challenge of managing HIV and NCDs in the elderly will continue to grow 33, 34. Careful thought needs to be given as to how best to reach and support this forgotten demographic in countries where HIV and youth are key health agenda items.

Three quarters of the HIV‐positive individuals in this sample had a BMI of greater than or equal to 25 kg/m^2^ (overweight or obese), whereas a quarter were hypertensive. The high prevalence of HTN in a similar community was also recently observed 35. An assessment of the impact of HIV comorbidities on morbidity and mortality in high HIV prevalence settings with limited resources is needed. Responding to these contemporary global health challenges requires a more complex and nuanced understanding of the problem. Addressing HIV, Malaria or Diarrhoeal disease in isolation misses the opportunity and need posed by the growing burden of non‐communicable disease present in these populations. The findings of this study underscore this fact showing the high prevalence of both HIV and non‐communicable disease and suggest that home‐based multi‐disease screening is feasible.

### 4.1 Relationship between HIV and NCDs

HIV association with CVD is possibly related to traditional CVD risk factors, HIV‐mediated inflammation and side effects of ART. Numerous studies have shown an increased morbidity and mortality of CVD endpoints among people living with HIV in developed countries 36, 37, 38. Interestingly, this study found relatively similar prevalence between HIV and non‐HIV persons for traditional CVD risk factors (excluding weight). US and European studies have found increased incidence of myocardial injury compared to non‐HIV comparison when controlling for these traditional risk factors 39, 40. Prevalence studies in Uganda found decreased rates of HTN among older people living with HIV, possibly related to improved follow‐up care 41. Unfortunately, in this study we did not collect prior and/or ongoing ART use and should be included in future studies. HIV‐infected patients on ART are at increased risk for fat redistribution and metabolic syndrome that could predispose this population to increased cardiometabolic disease risk 42.

### 4.2 Limitations

As a cross‐sectional study, this study has a number of limitations. The cross‐sectional design prevents the drawing of causal inferences. Data were collected from a small geographic area limiting the generalizability of the findings to other communities and population groups in South Africa. Although a small number of participants refused participation, a large number of participants from the original study were not contacted. It is feasible that healthier individuals were more likely to move, thereby potentially biasing this sample. Important medical history information was not collected from participants. Whether or not participants were taking ART, measurement of viral load and what other medication participants were taking was not collected. Finally, some of the methods of assessment were limited by resource constraints. For example blood pressure readings were collected only once. We measured blood pressure three times on one occasion but it would have been preferable to repeat these measures on two more separate occasion so as to define clinical HTN and reduce the impact of a white coat effect. Similarly, in addition to RPG it would have been preferable to also determine HbA1c concentrations to determine average blood sugar levels over the past period. Despite these limitations these data provide insight into the non‐communicable disease risk profile of people living in high HIV prevalence settings.

## 5 Conclusions

While limited in many ways, this study still shows clearly that a staggering burden of disease exists in this population. More effort is required to expand the breadth of community based health screening services beyond HIV to include NCDs and the risk factors that precede disease.

## Competing interests

The authors certify that they have NO affiliations with or involvement in any organization or entity with any financial interest, or non‐financial interest (such as personal or professional relationships, affiliations, knowledge or beliefs) in the subject matter or materials discussed in this manuscript

## Authors’ contributions

Conception of the work: A.V.H., R.B., H.V.R. Data collection: A.V.H. Data analysis and interpretation: A.V.H., R.B., S.A.N., L.M., H.V.R., C.C. Drafting the article: A.V.H. Critical revision of the article: R.B., H.V.R., S.A.N., L.M., C.C. Final approval of the version to be published: A.V.H., R.B., H.V.R., S.A.N., L.M., C.C.
